# Hepatoblastoma: when to screen?

**DOI:** 10.1186/1897-4287-9-S1-P30

**Published:** 2011-03-10

**Authors:** Margaret O’Malley, Lisa LaGuardia, Carol Burke, Matthew Kalady, James Church

**Affiliations:** 1 The Sanford R. Weiss, M.D. Center for Hereditary Colorectal Neoplasia, Digestive Diseases Institute, Desk A30, 9500 Euclid Ave., Cleveland Clinic,Cleveland, Ohio, 44195, USA

## Introduction

Hepatoblastoma is a rare hepatic malignancy that is seen in Familial Adenomatous Polyposis (FAP). The occurrence is four hundred fold higher than the general population [1]. Hepatoblastoma is generally treatable with surgery; in addition it is highly responsive to chemotherapy. While the literature reports on this extracolonic manifestation of FAP, no screening recommendations exist, this may be due to the rarity of the condition and paucity of published literature. Our aim was to examine and assess the number of hepatoblastoma patients, their associated demographics and the outcome of disease.

## Methods

Patients with FAP and hepatoblastomas were identified from a comprehensive polyposis database using Cologene© software. Variables that we assessed were age at diagnosis, hepatoblastoma characteristics, outcome, colonic / duodenal polyposis and management along with other extracolonics present.

## Result

Five patients, all male, from five families were diagnosed with hepatoblastomas in the last 23 years. The mean age at diagnosis was 17 months (+/^_^ 7 standard deviation). Genetic testing was available on 3/5 patients and were all 3 prime. No patient had a family history of hepatoblastoma, but 4/5 did have a family history of FAP. 4/5 presented with abdominal distention, tenderness or firmness. The youngest patient was diagnosed secondary to recurrent fevers. All patients had chemotherapy, 4/5 had resections of the hepatic tumor. 3/5 patients experienced total remission; one patient had two recurrences and a subsequent small bowel transplant due to desmoids disease. One underprivileged patient had late access to medical care due to poor socioeconomic status. He was diagnosed with an unresectable tumor and pulmonary metastasis disease, even after chemotherapy he died 6 months later. The median follow up time was 10 years (range 0.5 to 23). 2/5 patients have bilateral high frequency hearing loss from chemotoxicity resulting in the use of hearing aides. One patient had cardiac toxicity, he is being followed yearly. One patient suffered from short stature, pubertal delay secondary to chemotherapy.

## Conclusion

Our results are consistent with the published data on FAP. Patients with hepatoblastomas are generally young males with APC mutations in the 3 prime areas. This should prompt consideration of abdominal imaging in children with this constellation of genotype and hepatoblastoma demographics, regardless of family history. Further research into early detection of this possible mode of neoplasm is warranted.
